# External Tertiary-Care-Hospital Validation of the Epidemiological SEER-Based Nomogram Predicting Downgrading in High-Risk Prostate Cancer Patients Treated with Radical Prostatectomy

**DOI:** 10.3390/diagnostics13091614

**Published:** 2023-05-03

**Authors:** Cristina Cano Garcia, Mike Wenzel, Mattia Luca Piccinelli, Benedikt Hoeh, Lea Landmann, Zhe Tian, Clara Humke, Reha-Baris Incesu, Jens Köllermann, Peter J. Wild, Christoph Würnschimmel, Markus Graefen, Derya Tilki, Pierre I. Karakiewicz, Luis A. Kluth, Felix K. H. Chun, Philipp Mandel

**Affiliations:** 1 Department of Urology, University Hospital Frankfurt, Goethe University Frankfurt, 39120 Frankfurt am Main, Germany; 2 Cancer Prognostics and Health Outcomes Unit, Division of Urology, University of Montréal Health Center, Montreal, QC H2X 0A9, Canada; 3 Department of Urology, IEO European Institute of Oncology, Istituto Di Ricovero e Cura a Carattere Scientifico (IRCCS), 20141 Milan, Italy; 4 Martini-Klinik Prostate Cancer Center, University Hospital Hamburg-Eppendorf, 20246 Hamburg, Germany; 5 Dr. Senckenberg Institute of Pathology, University Hospital Frankfurt, 60590 Frankfurt am Main, Germany; 6 LOEWE Center Frankfurt Cancer Institute (FCI), Goethe University Frankfurt, 60439 Frankfurt am Main, Germany; 7 Department of Urology, Kantonspital Luzern, 6000 Luzern, Switzerland; 8 Department of Urology, University Hospital Hamburg-Eppendorf, 20246 Hamburg, Germany; 9 Department of Urology, Koc University Hospital, 34010 Istanbul, Turkey

**Keywords:** external validation, nomogram, downgrading, high risk, prostate cancer

## Abstract

We aimed to externally validate the SEER-based nomogram used to predict downgrading in biopsied high-risk prostate cancer patients treated with radical prostatectomy (RP) in a contemporary European tertiary-care-hospital cohort. We relied on an institutional tertiary-care database to identify biopsied high-risk prostate cancer patients in the National Comprehensive Cancer Network (NCCN) who underwent RP between January 2014 and December 2022. The model’s downgrading performance was evaluated using accuracy and calibration. The net benefit of the nomogram was tested with decision-curve analyses. Overall, 241 biopsied high-risk prostate cancer patients were identified. In total, 51% were downgraded at RP. Moreover, of the 99 patients with a biopsy Gleason pattern of 5, 43% were significantly downgraded to RP Gleason pattern ≤ 4 + 4. The nomogram predicted the downgrading with 72% accuracy. A high level of agreement between the predicted and observed downgrading rates was observed. In the prediction of significant downgrading from a biopsy Gleason pattern of 5 to a RP Gleason pattern ≤ 4 + 4, the accuracy was 71%. Deviations from the ideal predictions were noted for predicted probabilities between 30% and 50%, where the nomogram overestimated the observed rate of significant downgrading. This external validation of the SEER-based nomogram confirmed its ability to predict the downgrading of biopsy high-risk prostate cancer patients and its accurate use for patient counseling in high-volume RP centers.

## 1. Introduction

In prostate cancer, the discrepancy between the biopsy Gleason score and the radical prostatectomy (RP) Gleason score is a well-known phenomenon [1,2,3,4,5,6,7,8]. In particular, upgrading has been thoroughly studied in several single- and multi-institutional, as well as population-based databases [8,9,10,11,12]. Conversely, data that can be used to examine downgrading rates, particularly in biopsied high-risk prostate cancer patients, are limited due to the rarity of patients with Gleason scores ≥4 + 4 undergoing RP [13,14]. However, in prostate cancer patients undergoing RP, the prognosis and further treatment depend on the pathologic Gleason score of the RP sample in addition to surgical margins, pathological tumor (pT) stage, and lymph node invasion. Consequently, the risk of downgrading may affect treatment recommendations and patient counseling. Moreover, in prostate cancer patients who do not undergo RP, the biopsy Gleason score represents the only available sign of pathology. Consequently, this discrepancy between biopsy and RP Gleason scores may be clinically significant and may lead to over- or undertreatment in affected patients. Recently, our group developed a nomogram that predicts both downgrading and significant downgrading biopsied high-risk prostate cancer patients in the National Comprehensive Cancer Network (NCCN) treated with RP based on the Surveillance, Epidemiology and End Results (SEER) database [15]. Despite the rarity of the application of RP in prostate cancer patients at high = risk, or even very high risk, this SEER-based nomogram identified 6690 study patients. Consequently, population-based data repositories, such as the SEER, are essential to study rarer events, such as downgrading in high-risk and very-high-risk prostate cancer patients. However, this nomogram has not yet been externally validated, which is important, since the data from epidemiologic databases in the United States and from European tertiary-care hospitals with high patient volumes may differ. Therefore, we aimed to evaluate the performance of this nomogram in a contemporary external European tertiary-care-hospital cohort. This topic is of particular interest, since both epidemiologic and high-volume centers have shown an increased proportion of biopsied RP patients with high-risk prostate cancer in recent years [16,17,18].

## 2. Materials and Methods

### 2.1. Patient Population

**Figure 1:**
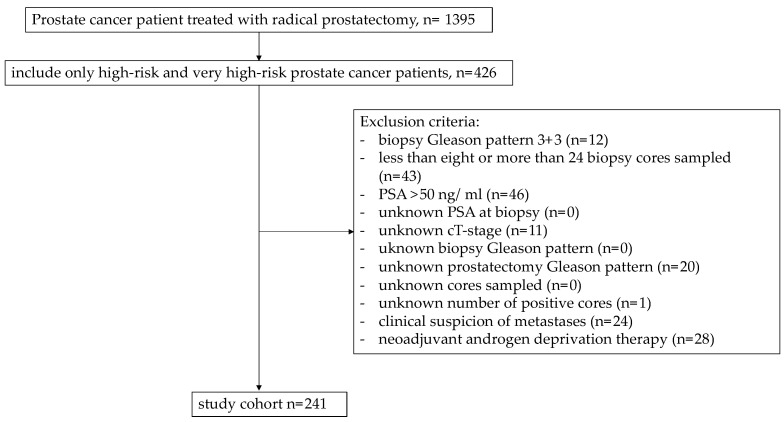
Consort flow diagram.

Our institutional prospectively collected prostate cancer database was used to retrospectively identify biopsy-confirmed patients in NCCN with high-risk prostate cancer (clinical tumor stage (cT) 3a or Gleason score 8–10 or prostate-specific antigen (PSA) > 20 ng/mL) treated with RP between January 2014 and December 2022 [19]. Only patients with biopsy Gleason pattern ≥3 + 4 were included, since downgrading is not applied at biopsy Gleason pattern 3 + 3. Moreover, based on the previous methodology of the initially published nomogram, only prostate cancer patients with eight to twenty-four biopsy cores sampled and PSA ≤ 50 ng/mL were included [20,21,22]. Exclusion criteria consisted of unknown PSA at biopsy, unknown cT stage, unknown biopsy Gleason pattern and unknown number of positive biopsy cores. Patients with neoadjuvant androgen-deprivation therapy (ADT) and clinical suspicion of metastases were also excluded (Figure 1). A biopsy Gleason score in each pathological stain was investigated, defined as the highest and worst Gleason pattern. Consequently, the highest Gleason score of all stains was defined as the biopsy Gleason score [23].

Ethical approval was obtained from the institutional review boards of the University Cancer Center Frankfurt (UCT) and the Ethical Committee at the University Hospital Frankfurt, and written informed consent was obtained from all patients.

### 2.2. Covariates and Study Endpoints

In the SEER-based nomogram predicting downgrading (relying on 6690 patients treated with RP between 2010 and 2016), covariables consisted of PSA at initial diagnosis (continuous variable), cT stage (cT1 vs. cT2 vs. cT3a vs. cT3b vs. cT4), number of positive biopsy cores (continuous variable), and biopsy Gleason pattern (3 + 4 vs. 4 + 3 vs. 4 + 5 vs. 4 + 4 vs. 5 + 4 vs. 5 + 3 vs. 3 + 5 vs. 5 + 5). In the SEER-based study, all these variables represented significant predictors of downgrading. According to the SEER-based nomogram, downgrading from biopsy to RP by at least one Gleason pattern represented the primary endpoint (henceforth referred to as “any downgrading”). Moreover, significant downgrading, defined as downgrading of primary or secondary Gleason pattern 5 at biopsy to ≤4 + 4 at RP, represented the secondary endpoint in subgroup analyses [15].

### 2.3. Statistical Analyses

Descriptive statistics were presented using frequency for categorical variables and median with interquartile range (IQR) for continuous variables. External validation was derived from the initial odds ratios (ORs) and intercepts of the above-mentioned covariates in the study by Wenzel et al. [15]. As recommended by the transparent reporting of a multivariable prediction model for individual prognosis or diagnosis (TRIPOD) statement, external validation of the predictive model was evaluated in terms of discrimination, calibration, and net benefit [24]. Discrimination was quantified using accuracy, as well as the area under the curve (AUC) and bootstrap 95% confidence interval (CI) from the receiver operating characteristic (ROC) curve. Calibration was assessed using calibration-in-the-large (CILR) and calibration slope. The extent of over-and underestimation was graphically described using calibration plots. Decision-curve-analysis (DCA) was used to evaluate the net benefit of the developed models. Finally, systematic analyses of several possible model probability cut-offs were performed. The R software environment for statistical computing and graphics (version 4.1.2) was used for all analyses [25].

## 3. Results

### 3.1. Descriptive Characteristics

Overall, 241 biopsied high-risk prostate cancer patients were identified and qualified for study inclusion. The median age and PSA at diagnosis were, respectively, 67 years (interquartile range [IQR] 63–72 years) and 10 ng/mL (IQR 6–21 ng/mL). The median number of prostate-biopsy cores taken at biopsy and median number of positive prostate-biopsy cores were, respectively 13 (IQR 12–14) and 6 (IQR 4–8). According to the NCCN risk category, 60% of the patients exhibited high-risk and 40% exhibited very high-risk prostate cancer at biopsy. Regarding the cT stage, highest rate was observed for cT2 (56%), followed by cT1c (35%), cT3a (5%), cT3b (3%). and cT4 (1%). According to the Gleason score at biopsy in the NCCN high-risk prostate cancer patients, the highest rate was observed for the Gleason score of 4 + 4 (39%), followed by 4 + 5 (24%), 4 + 3 (10%), 3 + 4 (9%), 3 + 5 (8%), 5 + 4 (6%), 5 + 5 (3%), and 3 + 5 (1%). The rate of any downgrading from biopsy to RP specimen was 51% in the overall cohort of 241 NCCN high-risk prostate cancer patients. In the subgroup of 99 patients with a primary or secondary biopsy Gleason pattern of 5, 43% were significantly downgraded from biopsy to RP Gleason pattern ≤ 4 + 4. When stratifying the study cohort for downgrading vs. no downgrading, the downgrading patients exhibited lower median PSA (8 vs. 14, *p* < 0.001) and frequently lower pT stages (pT2a: 5 vs. 2%, pT2b: 0 vs. 2%, pT2c: 35 vs. 19%, pT3a: 39 vs. 41%, pT3b: 22 vs. 34%, pT4: 0 vs. 3%, *p* = 0.004; Table 1).

### 3.2. External Validation of the SEER-Based Nomogram Predicting Any Downgrading in NCCN Biopsied High-Risk Prostate Cancer Patients

**Figure 2:**
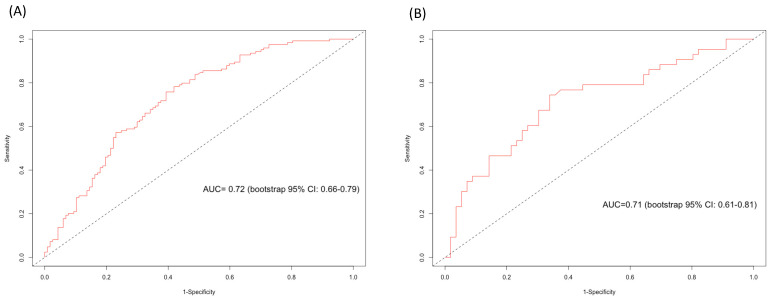
Area under the curve (AUC) and bootstrap 95% confidence interval (CI) on receiver operating characteristic (ROC) curves used to predict (**A**) any downgrading and (**B**) significant downgrading (any Gleason pattern 5 to Gleason pattern ≤ 4 + 4 at radical prostatectomy).

**Figure 3:**
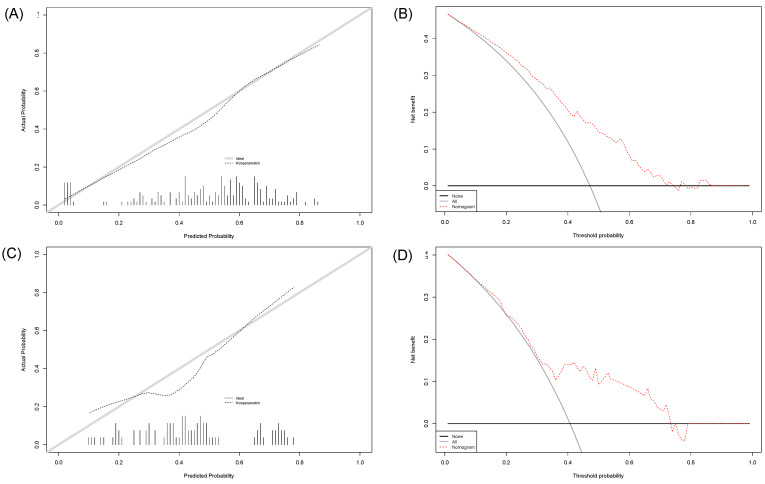
(**A**) Calibration plot illustrating the relationship between the observed rate of downgrading and predicted probability of downgrading for the main nomogram performance in the external validation cohort, on which the ideal relationship between the prediction and observed rate is depicted by the gray line. (**B**) Decision-curve analysis depicting the net benefit of the main nomogram in the external validation cohort (dotted red line), relative to random consideration of downgrading (black line) and the average rate of downgrading (gray line). The same is illustrated in (**C**,**D**) for the second nomogram predicting significant downgrading (any Gleason pattern 5 to Gleason pattern ≤ 4 + 4 at radical prostatectomy).

The published and now externally validated nomogram predicted any downgrading in our external high-volume tertiary-care RP cohort with 72% accuracy (AUC = 0.72, Figure 2A). On the calibration plot, a high agreement between the predicted and observed downgrading rates was observed across all the probabilities (Figure 3A). In the DCA, the use of the nomogram resulted in greater net benefit for the threshold probabilities between 0.50 and 0.75, relative to both competing strategies (treat no NCCN biopsied high-risk prostate cancer patients and treat all NCCN biopsied high-risk prostate cancer patients, Figure 3B).

### 3.3. Nomogram Cutoffs for Any-Downgrading Predictions

In Table 2 (part A) various nomogram cutoffs for any downgrading are displayed, according to the numbers and percentages of correctly classified patients (true positive) vs. those that were classified incorrectly (false positive) by the nomogram in our cohort. The predicted probabilities ranged from 1% to 80%. As suggested and discussed by Wenzel et al., the probability in the initial nomogram was 60% [15]. Using this suggested cutoff in the current cohort, we identified 78 patients (32.4%) who were above this cutoff, indicating a higher risk of any downgrading. Of these 78 patients, 55 (70.5%) exhibited any downgrading (true positive), while 23 (29.5%) did not exhibit any downgrading at RP (false negative). When a higher cutoff of 70% was used, 28 patients (11.6%) above the cutoff were identified. Of these 28 patients, 21 (75.0%) exhibited any downgrading (true positive), while 7 patients (25.0%) did not exhibit any downgrading at RP (false negative). Finally, when using a lower cutoff of 10%, 219 patients (90.9%) above the cutoff were identified. Of these 219 patients, 123 (56.2%) exhibited any downgrading (true positive), while96 (43.8%) did not exhibit any downgrading at RP.

### 3.4. External Validation of the SEER-Based Nomogram Predicting Significant Downgrading in NCCN Biopsied High-Risk Prostate Cancer Patients

The published and now externally validated nomogram predicted significant downgrading with 71% accuracy (AUC = 0.71, Figure 2B). On the calibration plot, deviations from the ideal predictions were noted for predicted probabilities between 30% and 50%; the nomogram overestimated the observed rate of significant downgrading in the probabilities outside this range (Figure 3C). In the DCA, the use of the nomogram resulted in a greater net benefit for threshold probabilities between 0.45 and 0.75, relative to both competing strategies (treat no NCCN high-risk prostate cancer patients or treat all NCCN high-risk prostate cancer patients, Figure 3D).

### 3.5. Nomogram Cutoffs for Significant-Downgrading Predictions

In Table 2 (part B) various nomogram cutoffs for significant downgrading are displayed, according to the numbers and percentages of correctly classified patients (true positive) vs. those who were classified incorrectly (false positive). The predicted probabilities ranged from 1% to 80%. The probability suggested by the initial nomogram developed by Wenzel et al. was 50%. In the current externally validated RP cohort, we identified 25 patients (25.3) who were above this suggested cutoff, indicating a higher risk of any downgrading. Of these 25 patients, 17 (68.0%) exhibited significant downgrading (true positive), while 8 (32.0%) did not exhibit significant downgrading at RP (false negative).

## 4. Discussion

The data that can be used to study downgrading, especially on biopsied patients in the NCCN with high-risk prostate cancer, are limited. Therefore, the SEER-based nomogram for the prediction of downgrading in biopsied high-risk prostate cancer patients in the NCCN treated with RP was developed by Wenzel et al. In the present study, we externally validated this nomogram within a contemporary, external European high-volume tertiary RP cohort and made several important observations.

First, we observed an overall downgrading rate of 51% in the current biopsied high-risk prostate cancer patients in the NCCN treated with RP. Moreover, significant downgrading was observed in 43% of the cases (from any Gleason pattern of 5 to Gleason scores ≤4 + 4). The downgrading rates in our study (51% for any downgrading and 43% for significant downgrading) were in agreement with the rates reported by Wenzel et al. (50% for any downgrading and 44% for significant downgrading) [15]. Previously reported downgrading rates in other tertiary-care-based RP cohorts of patients with Gleason grades of 4 ranged from 45% to 61.5% [26,27,28]. However, these rates cannot be directly compared to those in previous downgrading reports, since no other reports focused only on biopsied high-risk prostate cancer patients while also including Gleason grade group 5. Surprisingly, the downgrading rates reported with the initial nomogram derived from the epidemiological SEER database are not significantly different from our reported downgrading rates, even though the SEER database’s pathological reports do not undergo central pathological review. It may be hypothesized that, due to the known phenomenon of discrepancies in pathological biopsy-core reviews, the SEER-based rates may differ from our rates due to differences in pathologists’ experiences across United States registries.

Second, we made important observations when comparing the tumor characteristics of biopsied high-risk prostate cancer patients in the NCCn from the current European high-volume tertiary-care RP cohort with those of the SEER-based NCCN high-risk prostate cancer cohort. For example, important differences were observed in the patients with organ-confined disease. Specifically, the rate of cT1c was significantly lower in the current cohort than in the SEER-based cohort (35% vs. 52%). Conversely, the rate of cT2 was higher in the current cohort than in the SEER-based cohort (56% vs. 36%). However, the rates of clinical suspicion of non-organ-confined disease (≥ cT3) were comparable between the two cohorts (9% vs. 12%) and may be explained by the rarity of patients undergoing RP with ≥ cT3 on digital rectal examination due to the higher risk of surgical complications [29]. Moreover, we observed comparable biopsy-Gleason-score distributions in the current cohort and the SEER-based cohort. Specifically, a biopsy Gleason score 4 + 4 was the most frequent and 5 + 3 was the least frequent score in both cohorts. Conversely, in the RP-Gleason-score distribution, important differences were observed. Specifically, in the current cohort, the rate of RP Gleason scores of 4 + 5 was higher than in the SEER-based cohort (28% vs. 20%). Conversely, the rate of RP Gleason scores of 4 + 4 in the current cohort was lower (8% vs. 14%).

Third, the external validation of the SEER-based nomogram resulted in a 72% accuracy for any downgrading at RP. The external validation by Wenzel et al. resulted in a 71% accuracy for any downgrading [15]. Consequently, the externally validated accuracy based on our European tertiary-care-hospital cohort marginally exceeded the initial internally validated accuracy obtained by Wenzel et al. The external validation of the SEER-based nomogram predicting any downgrading is therefore clinically important, since it confirms that the nomogram is robust and may be generalizable to a European RP cohort with biopsied high-risk prostate cancer patients in the NCCN. Moreover, it indicates that an easily applicable nomogram with clinical characteristics can be accurately used to predict the likelihood of any downgrading. Specifically, the threshold of 60% initially suggested by Wenzel et al. seems adequate, as it identifies 32.4% of patients at risk of downgrading at final pathology and 70.5% of those truly downgraded at RP. Using the same cutoff of 60%, Wenzel et al. reported a lower rate of actually downgraded RP patients, of 67.5% [15].

Finally, the external validation of the SEER-based nomogram resulted in a 71% accuracy for significant downgrading. The external validation by Wenzel et al. resulted in a 68% accuracy for significant downgrading [15]. Consequently, the externally validated accuracy based on our European tertiary-care-hospital cohort exceeds the initial accuracy validated by Wenzel et al. It is noteworthy that in the current biopsied high-risk prostate-cancer-patient cohort, only 8% (*n* = 20) had a Gleason score of 3 + 5 and 1% (*n* = 1) had a score of 5 + 3. Consequently, most of the significant downgrading applied to the downgrading from Gleason-grade group 5 (Gleason scores of 4 + 5, 5 + 4, and 5 + 5). The current observations regarding significant downgrading could be even more important for patient consultations, since significant downgrading may influence treatment decisions, the prediction of the need for adjuvant radiotherapy, and post-RP management to an even greater extent than any downgrading at RP.

Taken together, the current results confirmed the frequent occurrence of downgrading in biopsied high-risk prostate cancer patients in the NCCN, with an any-downgrading rate of 51% and a significant-downgrading rate of 43% in a European high-volume tertiary-care RP center. Moreover, both differences and similarities in terms of tumor characteristics (cT-stage, biopsy, and RP Gleason score) of in the biopsied high-risk prostate cancer patients in the NCCN undergoing RP were observed between the European high-volume tertiary-care cohort and the epidemiological SEER-database cohort. However, the external validation of the SEER-based nomogram’s prediction of any downgrading and of significant downgrading resulted in good accuracy. Consequently, the generalizability of this nomogram may be suggested. For prostate cancer patients undergoing RP, prognosis and further treatment depend mainly on the Gleason score of the RP. Therefore, the risk of downgrading may affect treatment recommendations and patient counseling. In addition, a previous report found that downgrading was associated with a lower risk of biochemical recurrence [30].

Despite its novelty, the current study has limitations. First, we relied on a single-institution database with the retrospective inclusion of patients. Second, the biopsy and RP specimens may have differed across pathology institutes since some of the prostate cancer patients were referred to our center from outpatient urology practices. Third, there was heterogeneity in the definition of the biopsy Gleason score, which may have biased the downgrading rates. Finally, some of our analyses and cut-off predictions may have been limited by the sample size, especially for significant downgrading.

## 5. Conclusions

The external validation of the SEER-based nomogram confirmed its ability to predict downgrading and significant downgrading in biopsied high-risk prostate cancer patients treated with RP within our European high-volume cohort. These findings support the potential role of the nomogram in treatment decisions and patient counseling.

## Figures and Tables

**Table 1:** Baseline characteristics of 241 biopsied high-risk prostate cancer patients treated with radical prostatectomy.

Characteristic	Overall ^1^ *n* = 241 (100%)	Downgrading ^1^ *n* = 124 (51%)	No Downgrading ^1^ *n* = 117 (49%)	*p*-Value ^2^
Age at diagnosis (years)	67 (63, 72)	67 (62, 71)	67 (64, 72)	0.2
Diabetes mellitus	20 (8%)	8 (7%)	12 (10%)	0.3
Hypertension	87 (36%)	45 (36%)	42 (36%)	>0.9
PSA at diagnosis (ng/mL)	10 (6, 21)	8 (6, 12)	14 (7, 24)	<0.001
Number of prostate-biopsy cores	13 (12, 14)	13 (12, 14)	13 (12, 14)	0.14
Number of positive prostate-biopsy cores	6 (4, 8)	6 (4, 8)	6 (4, 8)	0.8
NCCN risk category				
High risk	144 (60%)	76 (61%)	68 (58%)	0.6
Very high risk	97 (40%)	48 (39%)	49 (42%)	
cT Stage				0.2
cT1	85 (35%)	42 (34%)	43 (37%)	
cT2	136 (56%)	76 (61%)	60 (51%)	
cT3a	11 (5%)	3 (2%)	8 (7%)	
cT3b	8 (3%)	3 (2%)	5 (4%)	
cT4	1 (1%)	0 (0%)	1 (1%)	
Gleason score at biopsy				
3 + 4	22 (9%)	1 (1%)	21 (18%)	
4 + 3	25 (10%)	6 (5%)	19 (16%)	
4 + 4	95 (39%)	59 (48%)	36 (31%)	
3 + 5	20 (8%)	15 (12%)	5 (4%)	
5 + 3	1 (1%)	0 (0%)	1 (1%)	
4 + 5	57 (24%)	27 (22%)	30 (26%)	
5 + 4	14 (6%)	10 (8%)	4 (3%)	
5 + 5	7 (3%)	6 (5%)	1 (1%)	
pT Stage				0.004
T2a	8 (3%)	6 (5%)	2 (2%)	
T2b	2 (1%)	0 (0%)	2 (2%)	
T2c	65 (27%)	43 (35%)	22 (19%)	
T3a	96 (40%)	48 (39%)	48 (41%)	
T3b	67 (28%)	27 (22%)	40 (34%)	
T4	3 (1%)	0 (0%)	3 (3%)	
Gleason score at radical prostatectomy				
3 + 3	5 (2%)	5 (4%)	0 (0%)	
3 + 4	62 (26%)	51 (41%)	11 (10%)	
4 + 3	66 (27%)	50 (40%)	16 (14%)	
4 + 4	18 (8%)	3 (2%)	15 (13%)	
3 + 5	6 (3%)	2 (2%)	4 (3%)	
5 + 3	2 (0.5%)	1 (1%)	1 (1%)	
4 + 5	68 (28%)	10 (8%)	58 (50%)	
5 + 4	13 (5%)	2 (2%)	11 (10%)	
5 + 5	1 (0.5%)	0 (0%)	1 (1%)	
Concordance				
Concordance	68 (29%)	-	68 (29%)	
Downgrading	124 (51%)	124 (51%)	-	
Upgrading	49 (20%)	-	49 (20%)	
Downgrading from any 5 + X to ≤ 4 + 4	-	43 (35%)	-	

^1^ Median (IQR = interquartile range); *n* (%), ^2^ Wilcoxon rank -um test; Pearson’s chi-square test; Fisher’s exact test. Abbreviations: PSA = prostate-specific antigen, NCCN = National Comprehensive Cancer Network, cT = clinical tumor stage, pT = pathological tumor stage. Due to rounding, the percentages may not add up to 100%.

**Table 2:** Analyses of nomogram cutoffs in (A) the cohort of 241 high-risk prostate cancer patients treated with radical prostatectomy (RP) predicting any downgrading between biopsy and RP and (B) the cohort of 99 high-risk prostate cancer patients treated with RP predicting significant downgrading from any biopsy-based primary or secondary Gleason pattern 5 to Gleason pattern ≤ 4 + 4 at RP.

**A: Nomogram Cut-Offs Predicting Any Downgrading between Biopsy and RP Gleason**			
**Cutoff**	**Number of Patients above Nomogram Cutoff (%)**	**Number of Downgraded Patients above Nomogram Cutoff ** **(True Positives) (%)**	**Number of Patients ** **above Nomogram Cutoff without Downgrading ** **(False Positives) (%)**
10	219 (90.9)	123 (56.2)	96 (43.8)
15	219 (90.9)	123 (56.2)	96 (43.8)
20	216 (89.6)	122 (56.5)	94 (43.5)
25	212 (88.0)	121 (57.1)	91 (42.9)
30	200 (83.0)	118 (59.0)	81 (41.0)
35	188 (78.0)	114 (60.6)	74 (39.4)
40	181 (75.1)	110 (60.8)	71 (39.2)
45	158 (65.6)	101 (63.9)	57 (36.1)
50	135 (56.0)	89 (65.9)	45 (34.1)
55	112 (46.5)	77 (68.8)	35 (31.2)
56	105 (43.6)	73 (69.5)	32 (30.5)
57	101 (41.9)	72 (71.3)	29 (28.7)
58	92 (38.2)	66 (71.7)	26 (28.3)
59	88 (36.5)	62 (70.5)	26 (29.5)
60	78 (32.4)	55 (70.5)	23 (29.5)
61	70 (29.0)	49 (70.0)	21 (30.0)
62	63 (26.1)	45 (71.4)	18 (28.6)
63	60 (24.9)	42 (70.0)	18 (30.0)
64	59 (24.5)	41 (69.5)	18 (30.5)
65	59 (24.5)	41 (69.5)	18 (30.5)
66	49 (20.3)	35 (71.4)	14 (28.6)
67	42 (17.4)	30 (71.4)	12 (28.6)
68	36 (14.9)	25 (69.4)	11 (30.6)
69	34 (14.1)	25 (73.5)	9 (26.5)
70	28 (11.6)	21 (75.0)	7 (25.0)
75	16 (6.6)	11 (68.8)	5 (31.2)
80	4 (1.7)	3 (75.0)	1 (25.0)
**B: Nomogram Cut-Offs Predicting Significant Downgrading from Any Gleason Pattern 5 to RP Gleason ≥4 + 4**			
**Cutoff**	**Number of Patients above Nomogram Cutoff (%)**	**Number of Downgraded Patients above Nomogram Cutoff ** **(True Positives) (%)**	**Number of Patients ** **above Nomogram Cutoff without Downgrading ** **(False Positives) (%)**
10	99 (100)	43 (43.4)	56 (56.6)
15	95 (96)	43 (45.3)	52 (54.7)
20	90 (90.9)	41 (45.6)	49 (54.4)
25	87 (87.9)	41 (47.1)	46 (52.9)
30	79 (79.8)	38 (48.1)	41 (51.9)
35	73 (73.7)	36 (49.3)	37 (50.7)
40	58 (58.6)	33 (56.9)	25 (43.2)
41	57 (57.6)	33 (57.9)	24 (42.1)
42	52 (52.5)	32 (61.5)	20 (38.5)
43	47 (47.5)	29 (61.7)	18 (38.3)
44	43 (43.4)	26 (60.5)	17 (39.5)
45	41 (41.4)	26 (63.4)	15 (36.6)
46	38 (38.4)	24 (63.2)	14 (36.8)
47	35 (35.4)	22 (62.9)	13 (37.1)
48	31 (31.3)	20 (64.5)	11 (35.5)
49	28 (28.3)	20 (71.4)	8 (28.6)
50	25 (25.3)	17 (68.0)	8 (32.0)
51	23 (23.2)	16 (69.6)	7 (30.4)
52	22 (22.2)	16 (72.7)	6 (27.3)
53	21 (21.2)	16 (76.2)	5 (23.8)
54	20 (20.2)	15 (75.0)	5 (25.0)
55	20 (20.2)	15 (75.0)	5 (25.0)
60	20 (20.2)	15 (75.0)	5 (25.0)
65	20 (20.2)	15 (75.0)	5 (25.0)
70	13 (13.1)	10 (76.9)	3 (23.1)
75	4 (4.0)	3 (75.0)	1 (25)
80	0 (0)	0 (0)	0 (0)

## Data Availability

The data presented in this study are available on request from the corresponding author.
